# Supplementary material for: Efficacy and safety evaluation of artificial intelligence-identified antimicrobial peptides targeting avian pathogenic Escherichia coli in broiler chickens
Source: J Anim Sci Biotechnol. 2026 May 15;17:93. doi: 10.1186/s40104-026-01417-8 (PMC13177867; doi:10.1186/s40104-026-01417-8)
Supplement: Supplementary file 1 — Additional file 1: Table S1. Antimicrobial peptide (AMP) and dosage tested on two chicken breeds. Table S2. Additional in vitro testing for select AMPs. Table S3. qPCR assay primer/probe sequences. Table S4. qPCR reaction conditions. Table S5. qPCR assay efficiencies by assay and tissue type. Table S6: Hatchability of eggs in APEC challenge trials. Fig. S1. Kaplan–Meier survival plots of chickens treated with 20 μg TeRu4, TeBi1, or PeNi4. Fig. S2. Kaplan–Meier survival plots of birds treated with either 1, 5, or 10 µg AMP. Fig. S3. Weight gain of chickens treated with TeRu4 and PeNi4. Fig. S4. APEC infection percentages in birds across multiple post-hatch time points and tissues. Fig. S5. Relative fold change of cytokine transcripts in the cecal tonsil of APEC challenged birds. Fig. S6. Relative fold change values of cytokine transcripts in the spleen of APEC challenged birds. Fig. S7. Relative fold change of IL-1β, IL-8, and IL-6 transcripts lipopolysaccharide (LPS)-treated HD11 cells. Fig. S8. Relative fold change of IL-1β, IL-8, and IL-6 transcripts AMP-preincubated HD11 cells. Fig. S9. Raw percentage values for hatchability parameters across various AMPs and dosages. Fig. S10. Kaplan–Meier curves survival plots of the bird sexes on d 10 of the pen trials. Fig. S11. Kaplan–Meier survival plots of control and treatment groups on d 10 of the pen trials. Fig. S12. Kaplan–Meier survival plots of the bird sexes on d 35 of the pen trials. Fig. S13. Kaplan–Meier survival plots of experimental groups on d 35 of the pen trials. Fig. S14. Mean weights of female and male birds during pen trials across different AMPs and dosages. Fig. S15. Feed conversion ratio (FCR) of male and female birds during pen trials. Fig. S16. Flock uniformity of female and male birds during pen trials across different AMPs and dosages. Fig. S17. Relative cytokine transcripts in the spleens of pen trial birds treated with 10 µg TeBi1. Fig. S18. Stability of TeRu4 and Tebi1 in sterile water over 14 [file 40104_2026_1417_MOESM1_ESM.docx]

**TABLE OF CONTENTS**

| **Table S1** | Antimicrobial peptide (AMP) and dosage tested on two chicken breeds |
| --- | --- |
| **Table S2** | Additional in vitro testing for select AMPs |
| **Table S3** | qPCR assay primer/probe sequences |
| **Table S4** | qPCR reaction conditions |
| **Table S5** | qPCR assay efficiencies by assay and tissue type |
| **Table S6** | Hatchability of eggs in APEC challenge trials |
| **Figure S1** | Kaplan-Meier survival plots of chickens treated with 20 μg TeRu4, TeBi1, or PeNi4 |
| **Figure S2** | Kaplan-Meier survival plots of birds treated with either 1, 5, or 10 µg AMP |
| **Figure S3** | Weight gain of birds treated with TeRu4 or PeNi4 |
| **Figure S4** | APEC infection percentages in birds across multiple post-hatch time points and tissues |
| **Figure S5** | Relative fold change of cytokine transcripts in the cecal tonsil of APEC challenged birds |
| **Figure S6** | Relative fold change values of cytokine transcripts in the spleen of APEC challenged birds |
| **Figure S7** | Relative fold change of *IL-1β*, *IL-8*, and *IL-6* transcripts in lipopolysaccharide (LPS)-treated HD11 cells |
| **Figure S8** | Relative fold change of *IL-1β*, *IL-8*, and *IL-6* transcripts in AMP-preincubated HD11 cells |
| **Figure S9** | Raw percentage values for hatchability parameters across various AMPs and dosages |
| **Figure S10** | Kaplan-Meier survival plots of the bird sexes on d 10 of the pen trials |
| **Figure S11** | Kaplan-Meier survival plots of control and treatment groups on d 10 of the pen trials |
| **Figure S12** | Kaplan-Meier survival plots of the bird sexes on d 35 of the pen trials |
| **Figure S13** | Kaplan-Meier survival plots of experimental groups on d 35 of the pen trials |
| **Figure S14** | Mean weights of female and male birds during pen trials across different AMPs and dosages |
| **Figure S15** | Feed conversion ratio (FCR) of female and male birds during pen trials |
| **Figure S16** | Flock uniformity of female and male birds during pen trials across different AMPs and dosages |
| **Figure S17** | Relative cytokine transcripts in the spleens of pen trial birds treated with 10 µg TeBi1 |
| **Figure S18** | Stability of TeRu4 and Tebi1 in sterile water over 14 d. |

**SUPPLEMENTARY TABLES**

**Table S1.** Antimicrobial peptide (AMP) and dosage tested on two chicken breeds.

| **AMP and Dosage Tested**​ | **Chicken Breed Used**​ |
| --- | --- |
| TeBi1 - 10​ µg | Ross 308AP​ |
| TeBi1 - 20​ µg (Hatch Only)​ | Ross 308AP​ |
| TeBi1 - 20​ µg | COBB 500​ |
| TeRu4 - 10​ µg | COBB 500​ |
| TeRu4 - 20​ µg | Ross 308AP​ |
| TeRu4 - 20​ µg (Hatch Only)​ | COBB 500​ |
| PeNi4 - 20​ µg | COBB 500​ |

**Table S2.** Additional in vitro testing for select AMPs.

| **AMP** | **EC 25922 MIC (μg/mL)** | **EC BAA-2469 MIC (μg/mL)** | **EC BAA-2340 MIC (μg/mL)** | **EC BAA-2471 MIC (μg/mL)** | **EC CPO-NDM MIC (μg/mL)** | **EC MCR/NDM MIC (μg/mL)** | **SL-29 CC50 (μg/mL)** |
| --- | --- | --- | --- | --- | --- | --- | --- |
| TeRu4 | 1 | 1 | 0.5 | 0.75 | 0.875 | 1 | - |
| TeBi1 | 2 | 1 | 1 | 1 | 1.5 | 2 | >128 |
| PeNi4 | 32 | 32 | 32 | 16 | 9 | 32 | >128 |

Minimum inhibitory concentrations (MICs) were determined against *E. coli* isolates, and 50% cytotoxic concentrations (CC50) were evaluated against the SL-29 cell line. *E. coli* strains 25922, BAA-2469, BAA-2340, BAA-2471, and the chicken embryonic fibroblast cell line SL-29 were purchased from the American Type Culture Collection (ATCC, Manassas, VA, USA). Clinical isolates CPO-NDM and MCR/NDM were provided by the British Columbia Centre for Disease Control (BCCDC, Vancouver, BC, Canada).

**Table S3.** qPCR assay primer/probe sequences.

| **Assay Group** | **Gene Target** | **Forward Primer** | **Reverse Primer** | **Probe** |
| --- | --- | --- | --- | --- |
| Mplex1/4 | *IL-1β* | CTTCGACATCAACCAGAA | CGACATGTAGAGCTTGTA | FAM-TGCTTCGTG-ZEN-CTGGAGTCACC-IABkFQ |
|  | *rpl-8* | CAACCATCAGGAGAGATG | CTGGGTCTTTAGTTTTCC | HEX-TCGGTCTCA-ZEN-TTGCTGCTCGG-IABkFQ |
|  | *rps-10* | GCTCGTTGATAAGAATGT | CAAATTGCTCTTTCACATA | Cy5-AGCCATGCA-TAO-GTCTCTGAAATCC-IAbRQSp |
| Mplex2/5 | *IL-8* | GTAGGACGCTGGTAAAGA | GGTGGATGAACTTAGAATGA | FAM-ACTGGCACC-ZEN-GCAGCTCATTC-IABkFQ |
|  | *IL-10* | CCAGGTCAAAGAGAGTTA | CAGCAGCCTTTAAATCAA | HEX-TCCTATTAG-ZEN-AACCAACTGCTTAACTGCT-IABkFQ |
| Individual | *IFN-γ* | GTGGACCTATTATTGTAGAGA | GATGCTGAAGAGTTCATTC | FAM-CGCTGGATT-ZEN-CTCAAGTCGTTCAT-IABkFQ |
| Individual | *IL-6* | AGTGCCTGAATGTTTTAG | ACCGTAAGAAATGTAACAG | Cy5-CAATCCTCT-TAO-GTTACCAATCTGCCAC-IAbRQSp |
| Individual | *IRF-4* | AGGTGACAACTTCTAGTC | CTCAGCAGCTTTTCTATG | FAM-CTTCCTCTG-ZEN-GCTGTTATCCTCTGG-IABkFQ |

All sequences are 5' → 3'. Cy5, Cy5 fluorophore; FAM, FAM fluorophore; HEX, fluorophore; IABkFQ, Iowa Black FQ modified quencher; IAbRQSp, Iowa Black Rq Sp quencher; *IFN*, interferon; *IL,* interleukin; *IRF*, interferon regulatory factor; Mplex, multiplex reaction; *rps*, ribosomal protein small subunit encoding; TAO, TAO internal quencher; ZEN, ZEN internal quencher.

**Table S4.** qPCR reaction conditions.

| Tissue/Cell | Assays | Thermocycle temperature sequence (°C) | Time sequence (s) | Enzyme Mix | Primer^1^/Probe Concentration (nM) |
| --- | --- | --- | --- | --- | --- |
| Air sac, cecal tonsil, spleen | Mplex1, Mplex2, *IFN-γ, IRF-4* | 95, 56.5, 72 | 45, 30, 45 | Qiacuity Probe | 300/100 |
| HD11 cell line | Mplex4, Mplex5, *IL-6* | 95, 60, 72 | 15, 30, 30 | Sensifast Probe | 700/100 |

^1^Per primer

All assays use hot-start enzymes and are subject to an initial denaturation consisting of 9 minutes at 95 °C. All assays run for 45 cycles. Qiacuity Probe (QIAcuity Probe PCR Kit; Qiagen, Mississauga, ON, Canada); Sensifast Probe (Sensifast Probe No-ROX kit; FroggaBio, Concord, ON, Canada); see Supplementary Table S3 for other definitions.

**Table S5.** qPCR assay efficiencies by assay and tissue type. See Supplementary Table S3 for definitions.

| Tissue | Assay Group | Gene Target | Efficiency |
| --- | --- | --- | --- |
| Air sac | Mplex1 | *IL-1β* | 92.82% |
|  |  | *rpl-8* | 102.52% |
|  |  | *rps-10* | 102.70% |
|  | Mplex2 | *IL-8* | 107.52% |
|  |  | *IL-10* | 107.86% |
|  | Individual | *IFN-γ* | 97.51% |
|  | Individual | *IRF-4* | 98.01% |
| Cecal tonsil | Mplex1 | *IL-1β* | 93.69% |
|  |  | *rpl-8* | 98.01% |
|  |  | *rps-10* | 95.41% |
|  | Mplex2 | *IL-8* | 102.70% |
|  |  | *IL-10* | 101.78% |
|  | Individual | *IFN-γ* | 91.99% |
|  | Individual | *IRF-4* | 106.86% |
| Spleen | Mplex1 | *IL-1β* | 91.50% |
|  |  | *rpl-8* | 106.86% |
|  |  | *rps-10* | 99.42% |
|  | Mplex2 | *IL-8* | 90.98% |
|  |  | *IL-10* | 103.00% |
|  | Individual | *IFN-γ* | 86.88% |
|  | Individual | *IRF-4* | 90.44% |
| HD11 cells | Mplex4 | *IL-1β* | 84.32% |
|  |  | *rpl-8* | 86.08% |
|  |  | *rps-10* | 88.94% |
|  | Mplex5 | *IL-8* | 100.80% |
|  |  | *IL-10* | 98.44% |
|  | Individual | *IL-6* | 91.05% |

**Table S6**. Hatchability of eggs in APEC challenge trials.

| **Peptide** | **Dose (μg)** | **% Hatched** | **Fisher’s Exact Test Statistic (p-value)** |
| --- | --- | --- | --- |
| TeRu4 | 0 | 93% | N/A |
|  | 1 | 93% | 1 (1) |
|  | 5 | 95% | 0.7 (1) |
|  | 10 | 93% | 1 (1) |
| TeBi1 | 0 | 96% | N/A |
|  | 1 | 91% | 2.6 (0.4) |
|  | 5 | 95% | 1.5 (1) |
|  | 10 | 91% | 2.6 (0.4) |
| PeNi4 | 0 | 96% | N/A |
|  | 1 | 94% | 1.5 (1) |
|  | 5 | 90% | 2.6 (0.4) |
|  | 10 | 94% | 1.5 (1) |

Between 50-57 fertilized broiler chicken eggs were injected with a dose of peptide or PBS control on d 18 of incubation. The hatchability of eggs on d 21 was monitored and compared with Fisher’s Exact Test. There were no significant differences in hatchability between treated eggs and control eggs.


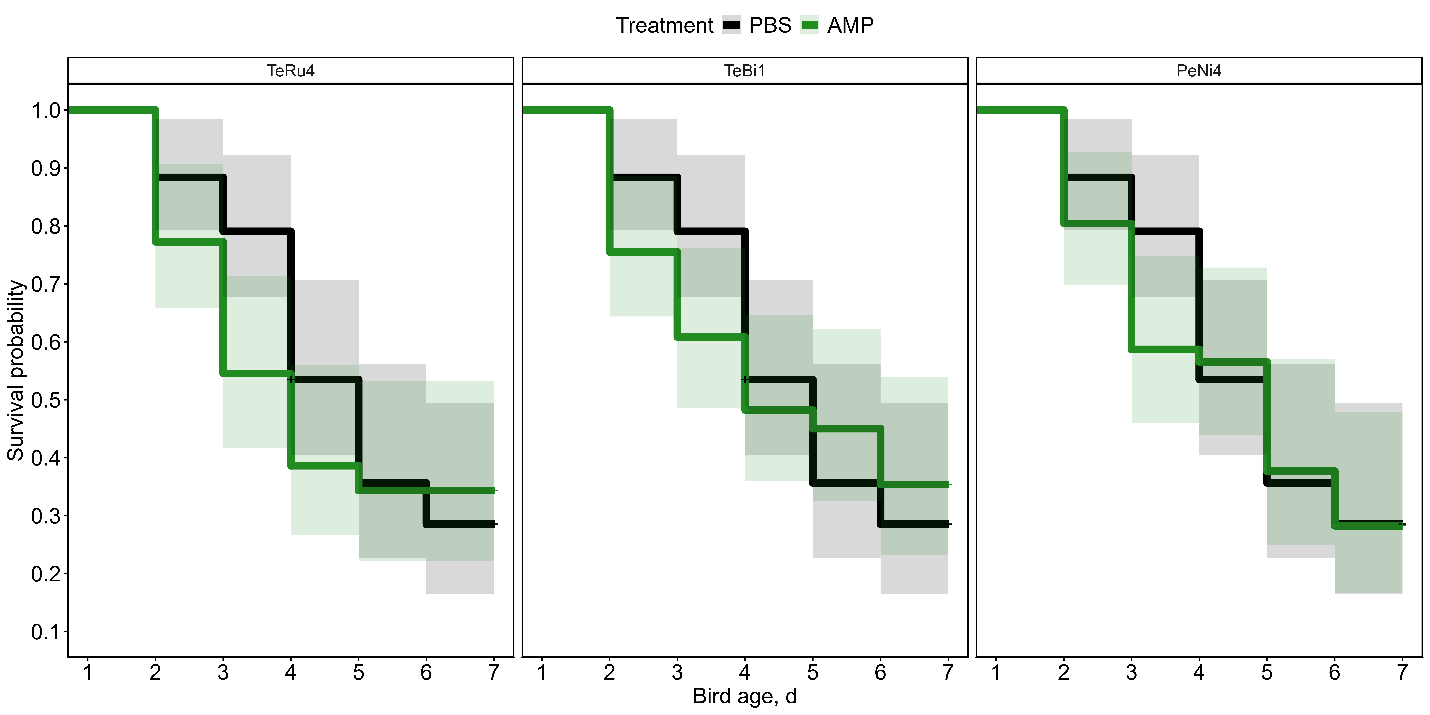
**Figure S1.** **Kaplan-Meier survival plots of chickens treated with 20 μg TeRu4, TeBi1, or PeNi4.** While cumulative survival by d 7 did not differ significantly between groups, mortality occurred earlier in some treated groups during the first 2-3 d post-challenge, prompting dose reduction in subsequent trials.


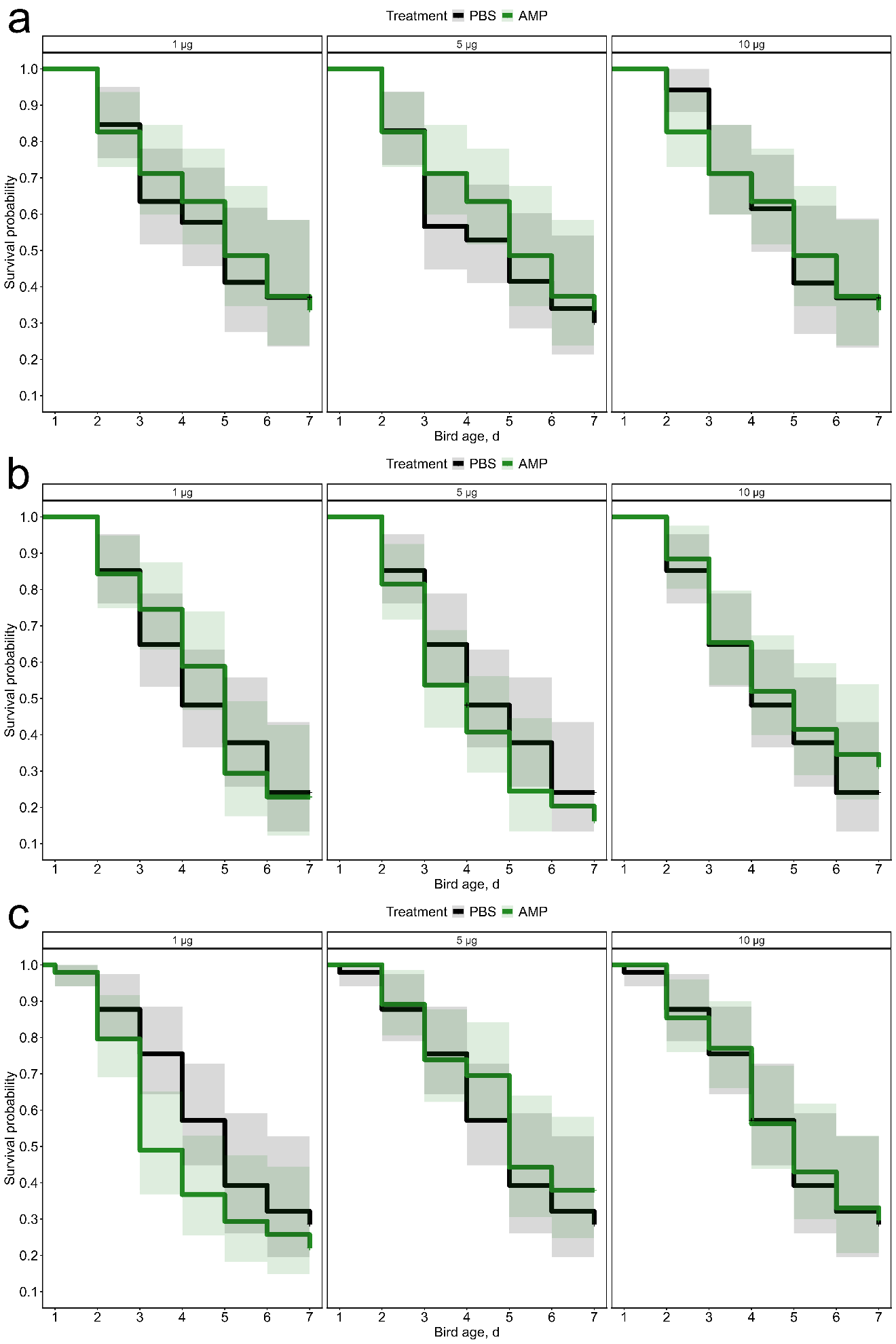


**Figure S2. Kaplan-Meier survival plots of birds treated with either 1, 5, or 10 µg AMP.** Plots shown are for a) TeRu4, b) TeBi1, or c) PeNi4. There were no significant differences in survival between treatment groups at any dose tested.


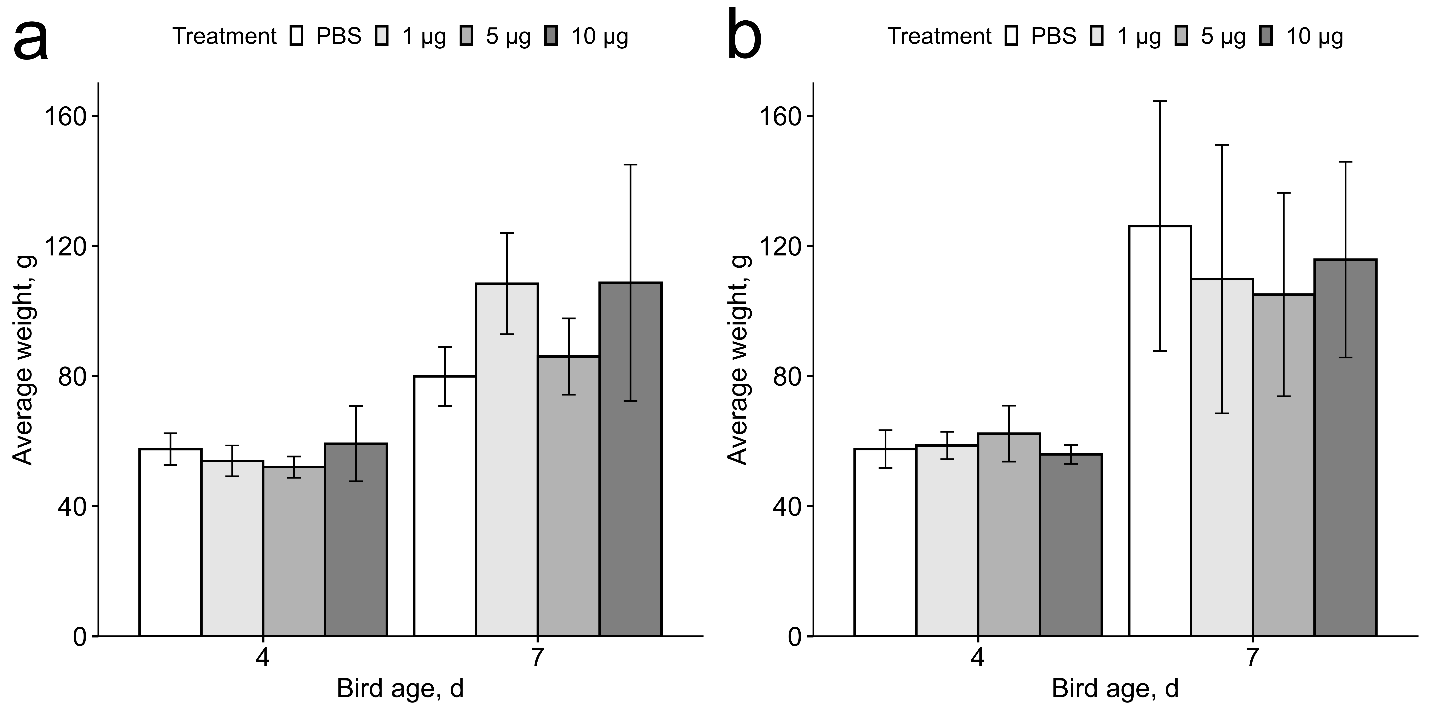


**Figure S3. Weight gain of birds treated with TeRu4 or PeNi4.** Plots shown are for a) TeRu4 and b) PeNi4 from d 4 to d 7 compared to their respective PBS controls. Birds were weighed individually and the values shown are median ± median absolute deviation (MAD). There were no significant differences in bird weight with treatment.


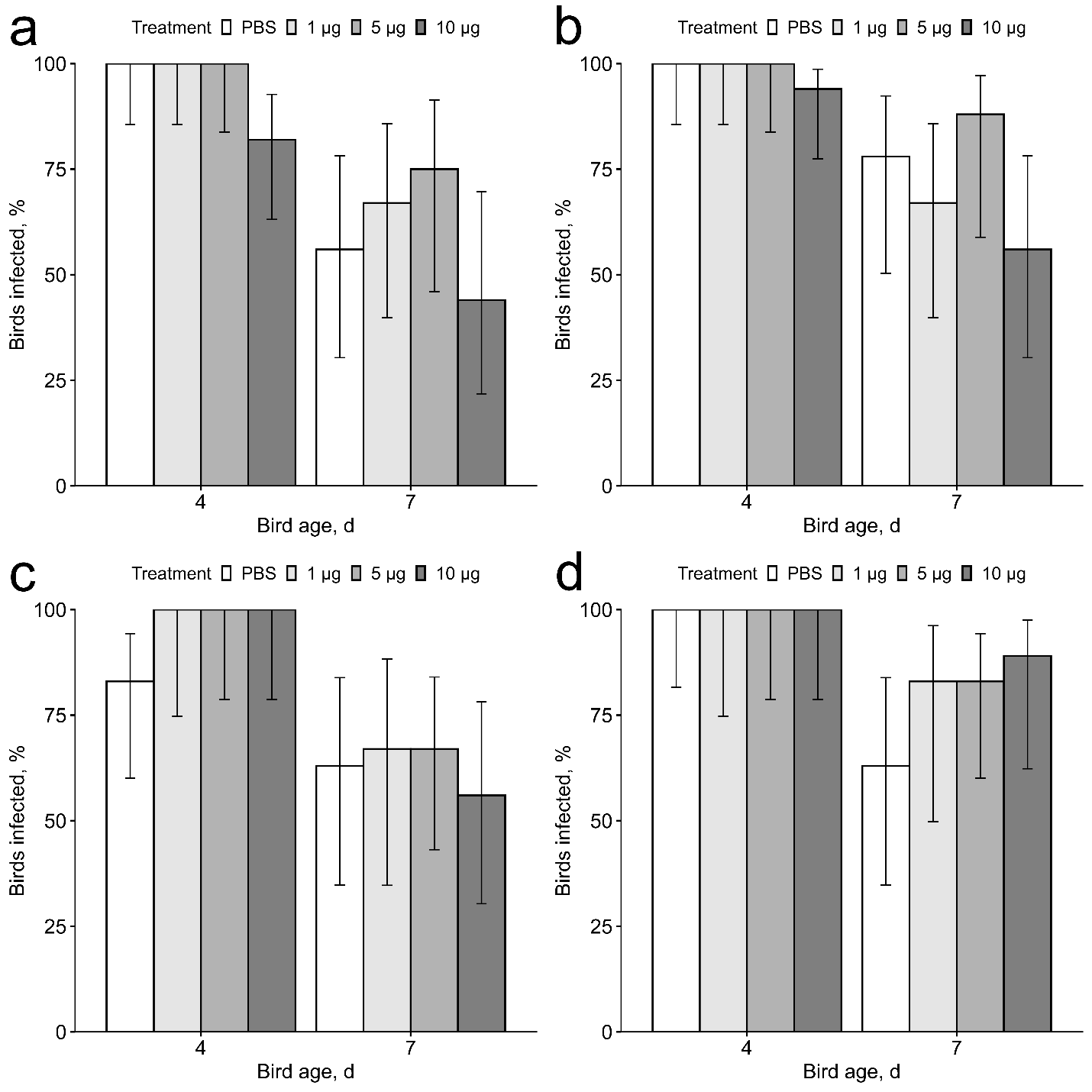


**Figure S4. APEC infection percentages in birds across multiple post-hatch time points and tissues.** Birds were treated with of 1, 5, or 10 µg of TeRu4 and PeNi4, compared against a PBS control: a) TeRu4, air sac; b) TeRu4, pericardium; c) PeNi4, air sac; d) PeNi4, pericardium. Birds were euthanized as scheduled and aseptic tissue swabs were streaked onto MacConkey agar. The percentage of swabs in each treatment group that resulted in bacterial growth is shown, with the error bars denoting a 90% Wilson confidence interval. Each treatment group was compared to the PBS control by Fisher’s Exact Test. No significant differences were observed between treatments.


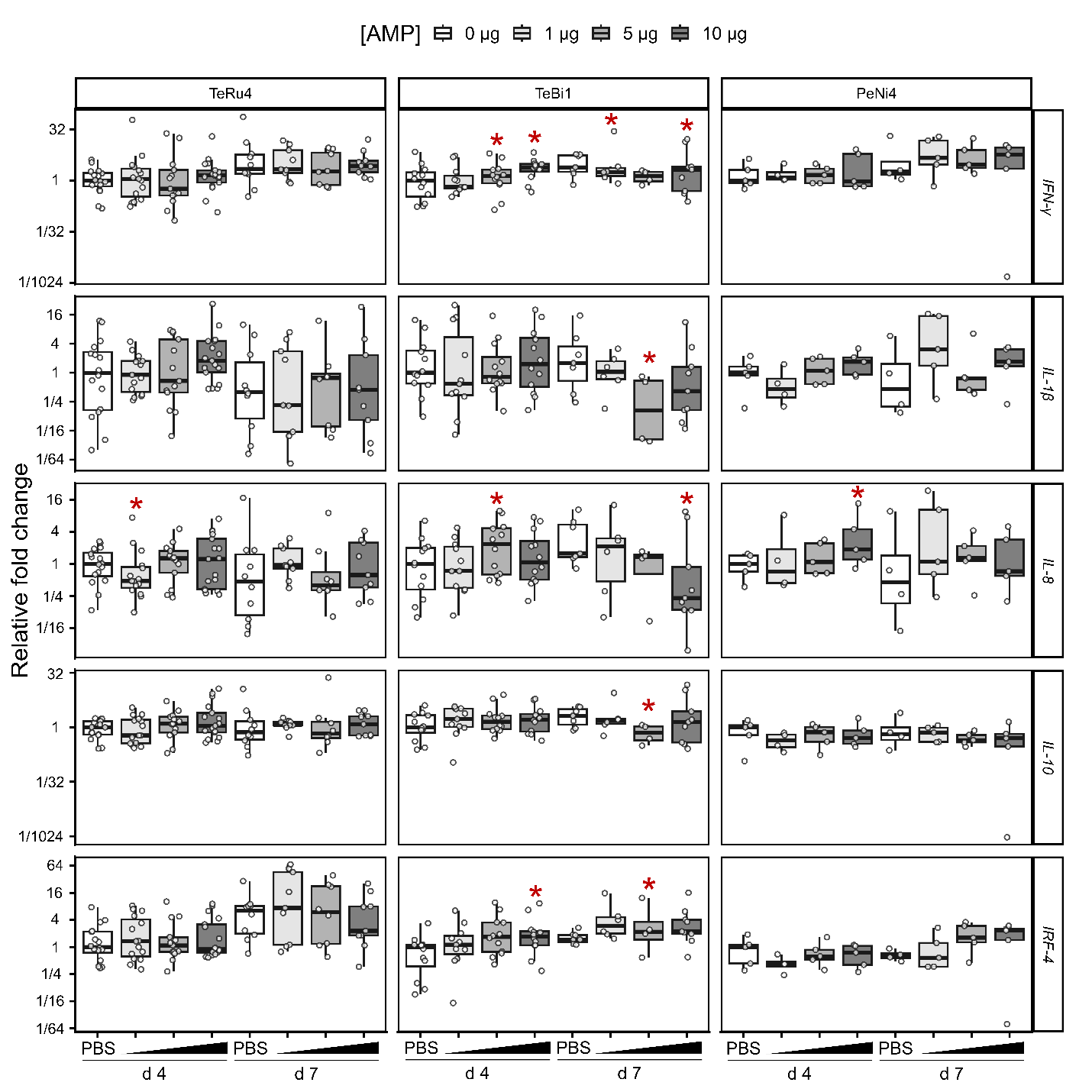


**Figure S5.** **Relative fold change of cytokine transcripts in the cecal tonsil of APEC challenged birds.** Birds were treated before hatch with 1, 5, or 10 μg AMP and euthanized on d 4 or 7 after hatch, and all values are relative to the PBS median on their respective days. Asterisks indicate significance at p ≤ 0.1. *IFN*, interferon; *IL,* interleukin; *IRF*, interferon regulatory factor.


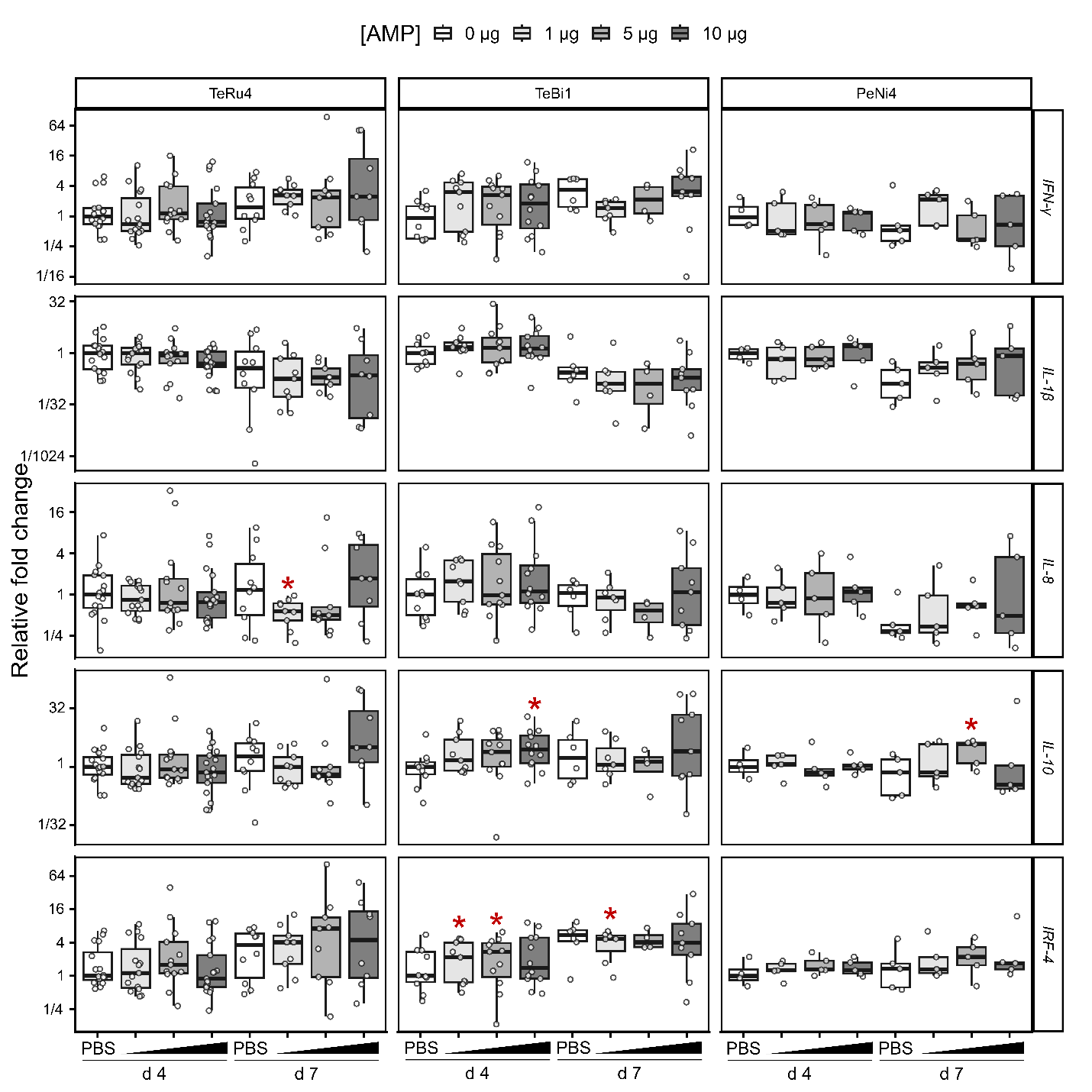


**Figure S6. Relative fold change values of cytokine transcripts in the spleen of APEC challenged birds.** Further details are in the Supplementary Figure S5 legend.


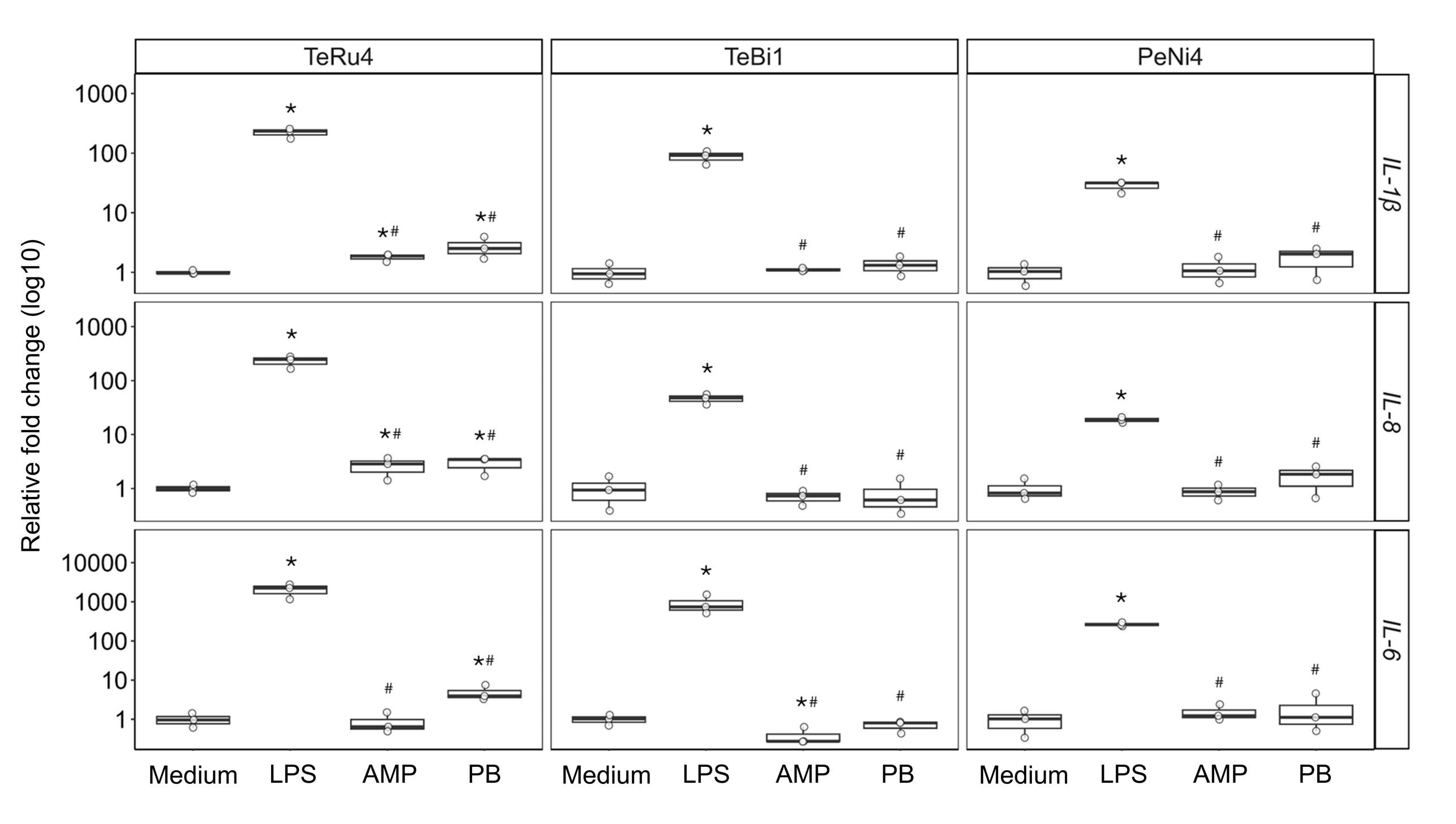


**Figure S7.** **Relative fold change of *IL-1β*, *IL-8*, and *IL-6* transcripts in lipopolysaccharide (LPS)-treated HD11 cells.** Cells were incubated for 6 h with medium, 25 ng/mL LPS, 16 µg/mL antimicrobial peptide (AMP; TeRu4, TeBi1, or PeNi4), or 8 µg/mL polymyxin B sulfate (PB) as determined by qPCR. A significant difference from the medium control is indicated by “*”, and “#” indicates a significant difference from LPS treatment (p-value < 0.1, Mann-Whitney-U test). Open circles represent the fold change of one technical replicate relative to the medium control. The horizontal lines represent the median of the three technical replicates within each treatment condition, the boxes show the interquartile range, and the vertical whiskers represent the minimum and maximum values. Shown are representative experiments of three biological replicates.





**Figure S8. Relative fold change of *IL-1β*, *IL-8*, and *IL-6* transcripts in AMP-preincubated HD11 cells.** Cells were incubated for 3 h with medium, a range of 2-fold serially diluted antimicrobial peptide (AMP; 1 to 16 µg/mL TeRu4, TeBi1, or PeNi4), or 8 µg/mL polymyxin B sulfate (PB), followed by the addition of 25 ng/mL LPS for an additional 3 h. Additional details are available in the Supplementary Figure S7 legend.


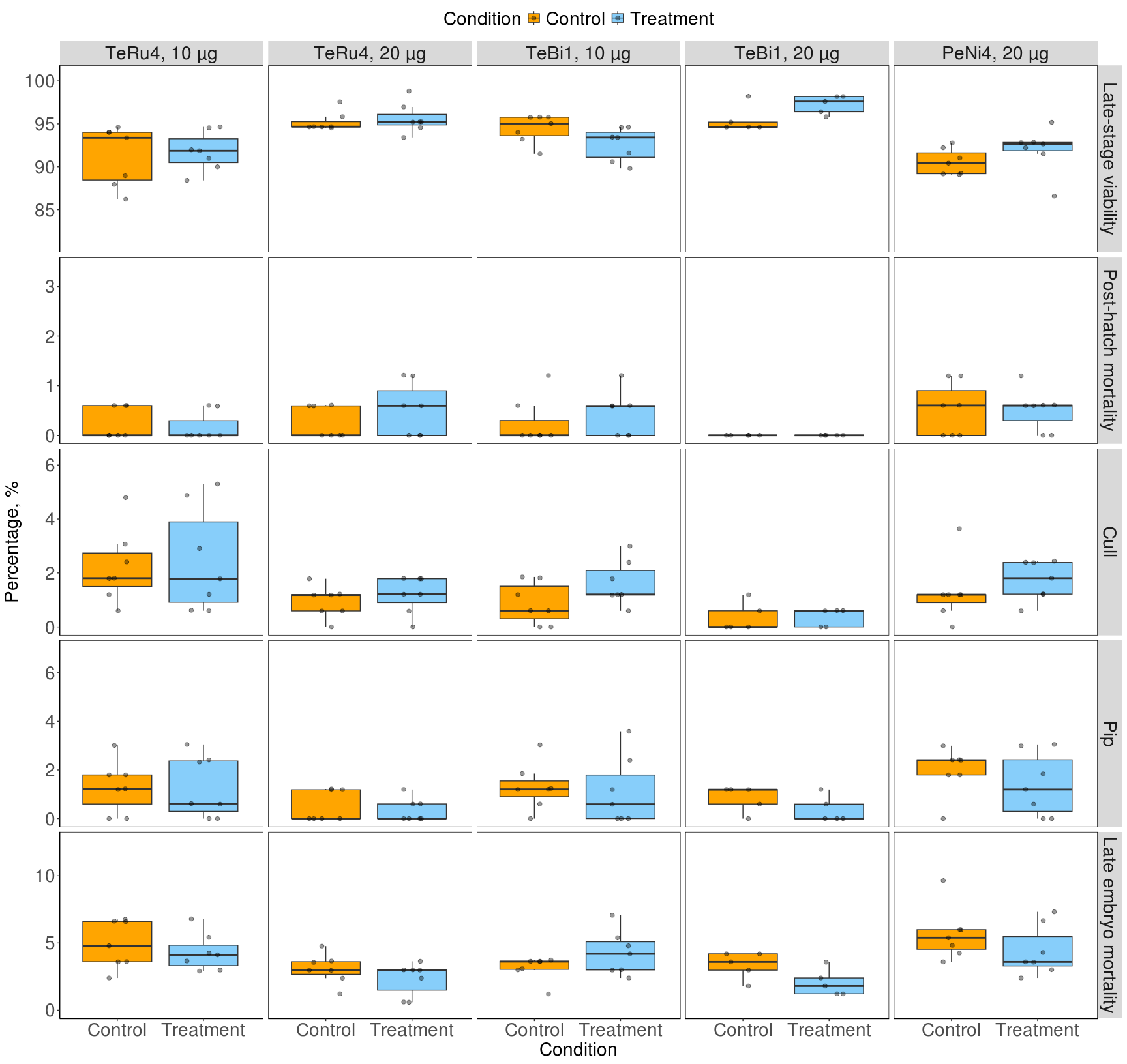


**Figure S9. Raw percentage values for hatchability parameters across various AMPs and dosages.** Columns represent AMPs at specific dosages, and rows represent individual hatchability parameters. Each AMP–dosage combination corresponds to a separate pen trial. The control (orange) and AMP treatment (blue) groups are shown. Each point corresponds to a tray of eggs (n = 7 per group; n = 5 for 20 µg TeBi1). The horizontal lines represent the median of the three technical replicates within each treatment condition, the boxes show the interquartile range, and the vertical whiskers represent the minimum and maximum values. No significant differences were detected using Wilcoxon rank-sum tests.


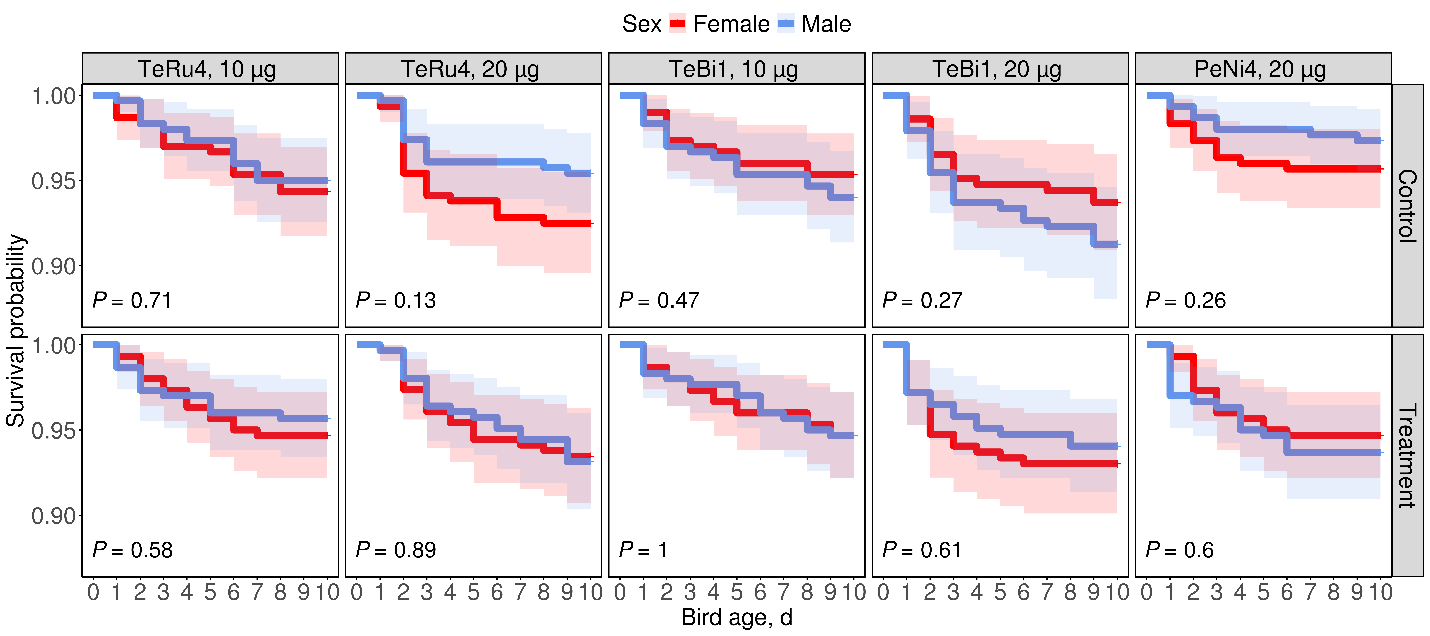


**Figure S10.** **Kaplan-Meier survival plots of the bird sexes on d 10 of the pen trials.** Here, survival probabilities on d 10 during the pen trials across different AMPs and dosages with sexes separated (female, red line; male, blue line) were calculated. Columns represent AMPs at specific indicated dosages, and rows distinguish between control and treatment groups. Each panel represents a separate pen trial. P-values, as determined by log-rank tests, are displayed in the bottom-left corner of each panel.


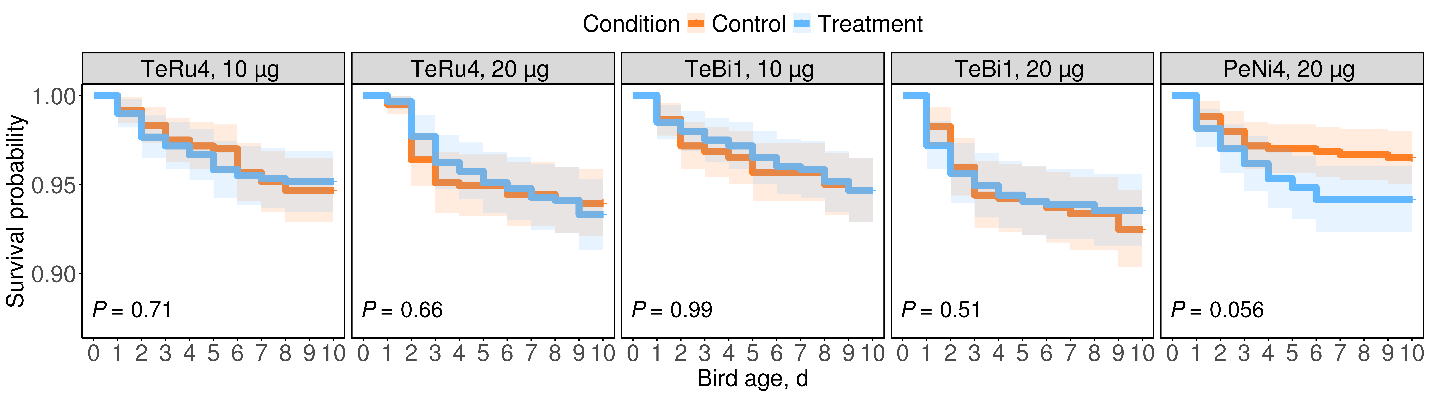


**Figure S11. Kaplan-Meier survival plots of control and treatment groups on d 10 of the pen trials.** Here, survival probabilities on d 10 during the pen trials across different AMPs and dosages were calculated with sexes combined as sexes were not significantly different from one another. The control (orange) and AMP treatment groups (blue) are shown. Each panel represents a separate pen trial. P-values, as determined by log-rank tests, are displayed in the bottom-left corner of each panel.


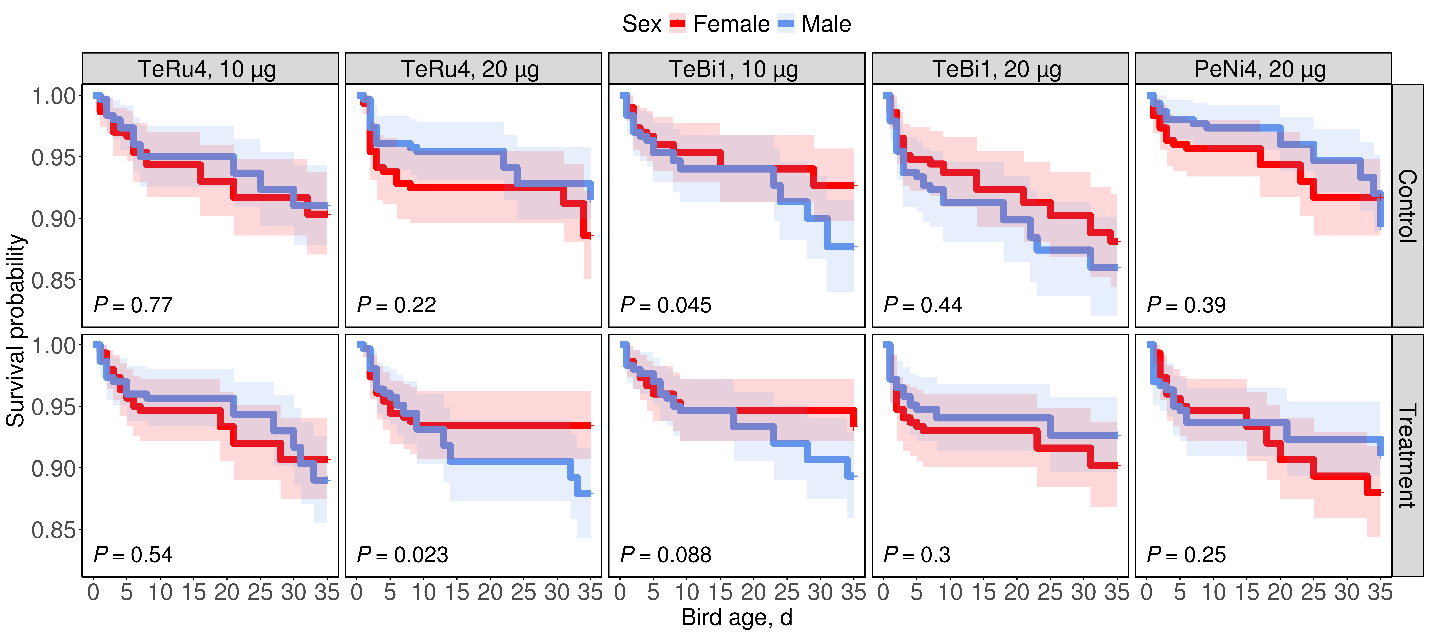


**Figure S12. Kaplan-Meier curves survival plots of the bird sexes on d 35 of the pen trials.** Additional details can be found in Supplementary Figure S10 legend.


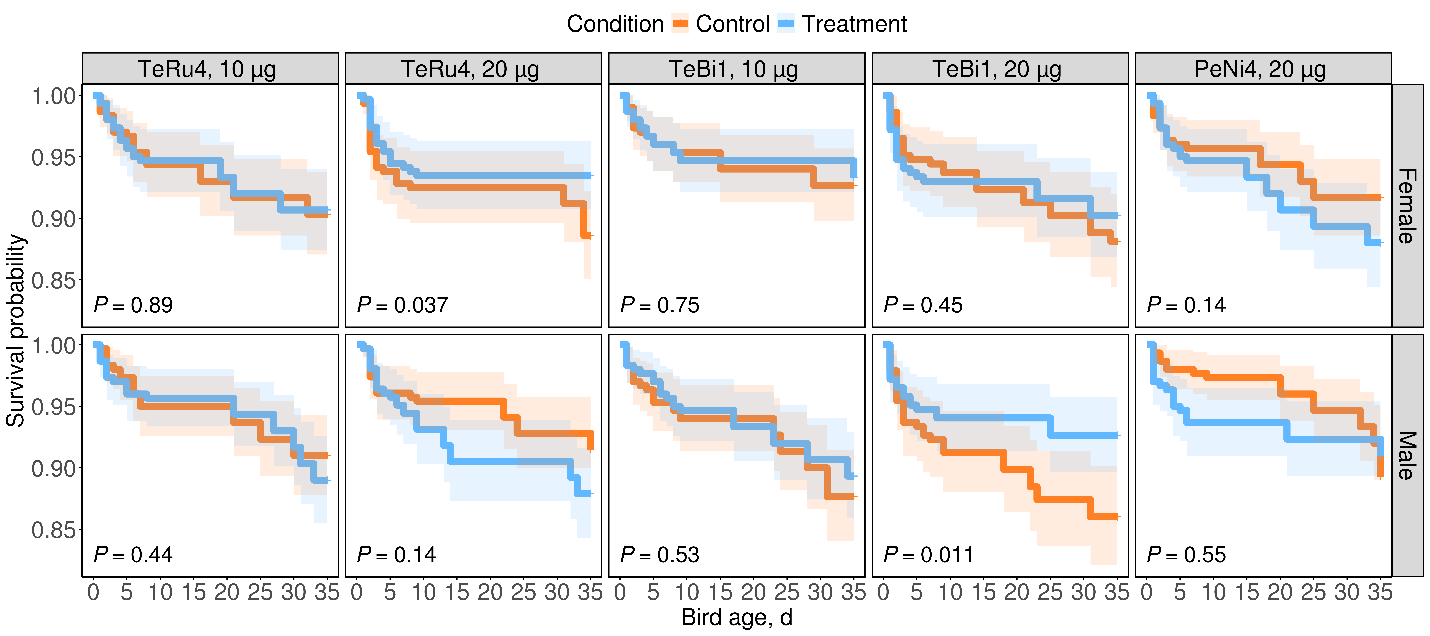


**Figure S13. Kaplan-Meier survival plots of experimental groups on d 35 of the pen trials.** Here, survival probabilities on d 35 during the pen trials across different AMPs and dosages were calculated with sexes separated as the survival probabilities of sexes were significantly different from one another in some trials. Additional details can be found in Supplementary Figure S11 legend.


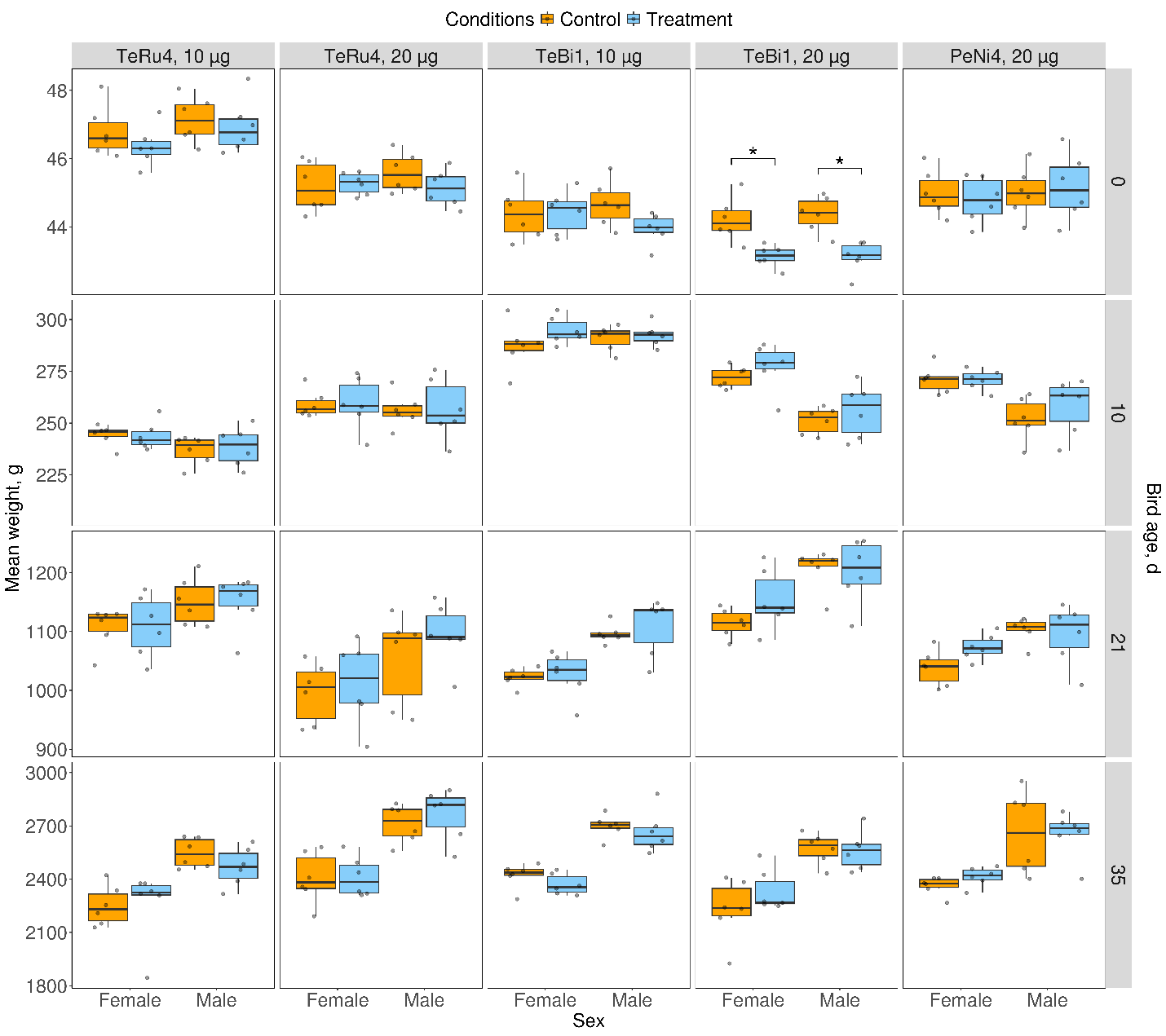


**Figure S14.** **Mean weights of female and male birds during pen trials across different AMPs and dosages.** The control (orange) and AMP treatment groups (blue) are shown. Each point represents the mean weight for a single mini-pen (n = 6 mini-pens per group). Significant differences (p<0.05) between control and treatment groups within a sex, as determined by Wilcoxon rank-sum tests, are indicated by an asterisk. Additional details can be found in the Supplementary Figure S9 legend.


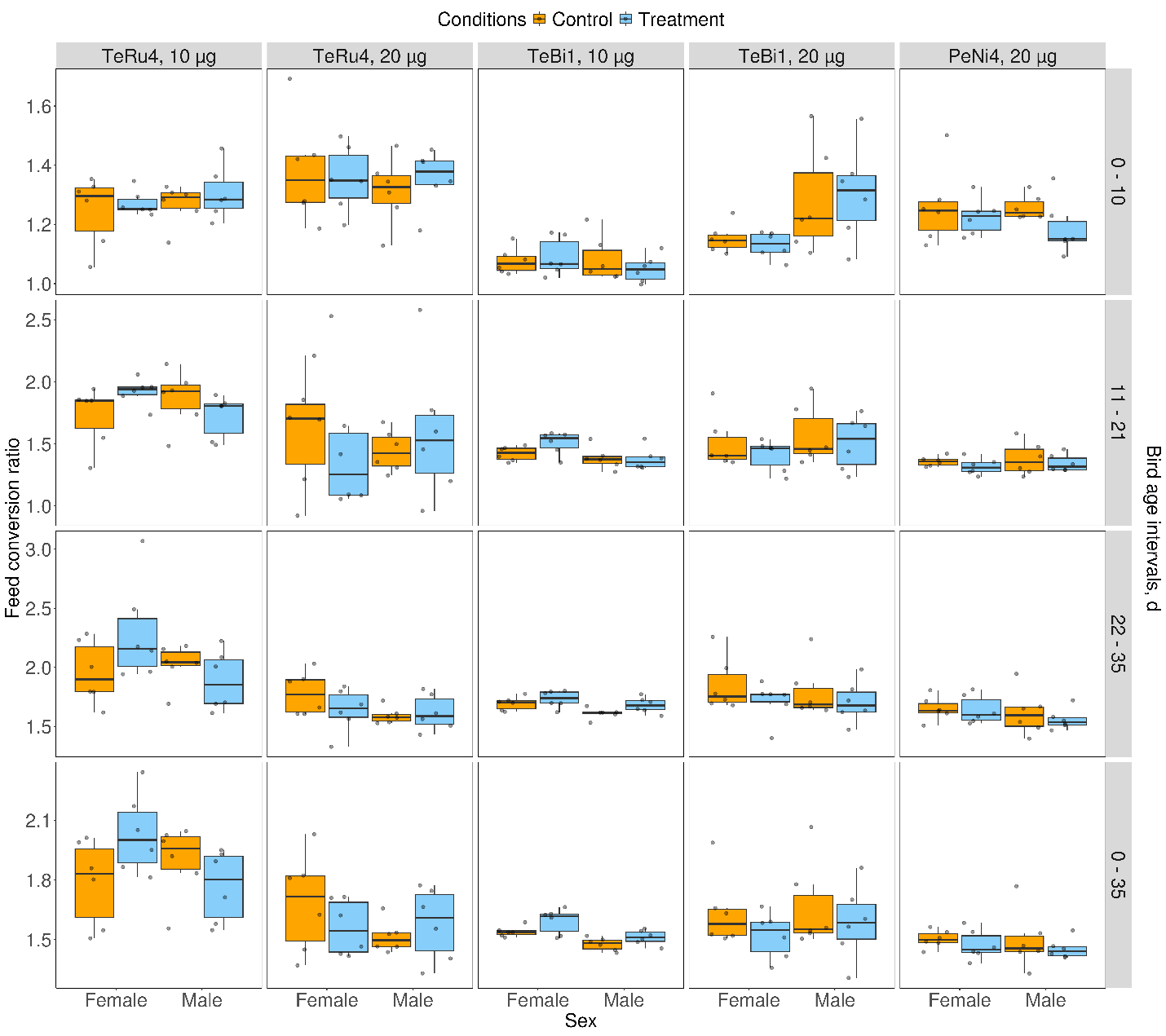


**Figure S15.** **Feed conversion ratio (FCR) of female and male birds during pen trials.** The control (orange) and AMP treatment groups (blue) are shown. Each point represents the FCR for a single mini-pen (n = 6 mini-pens per group). No significant differences were detected using Wilcoxon rank-sum tests. Additional details can be found in the Supplementary Figure S9 legend.


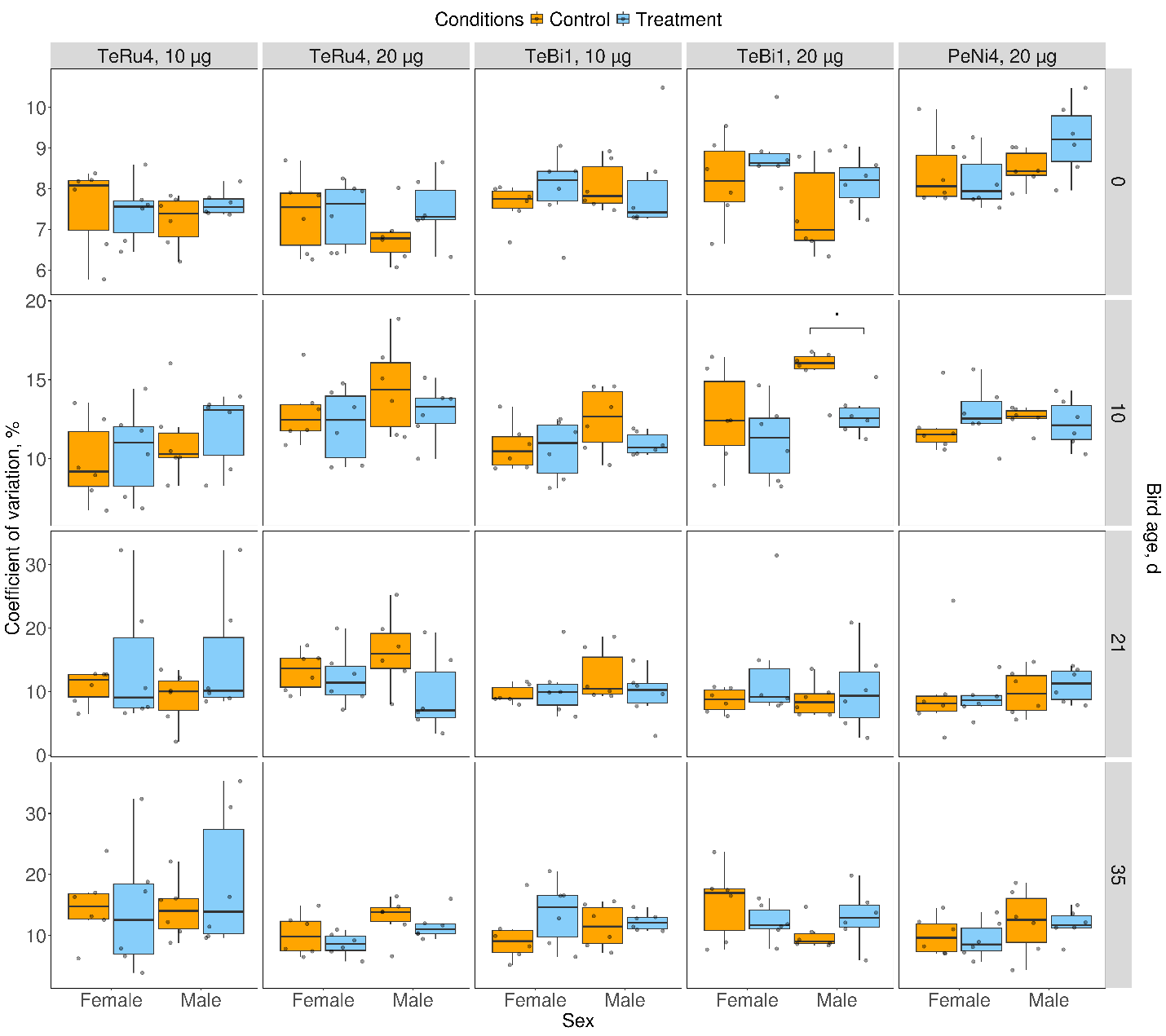


**Figure S16.** **Flock uniformity of female and male birds during pen trials across different AMPs and dosages.** The control (orange) and AMP treatment groups (blue) are shown. Each point represents the % CV for a single mini-pen (n = 6 mini-pens per group). Trends (0.05<p<0.1) between control and treatment groups within a sex, as determined by Wilcoxon rank-sum tests, are indicated by a dot. Additional details can be found in the Supplementary Figure S9 legend.


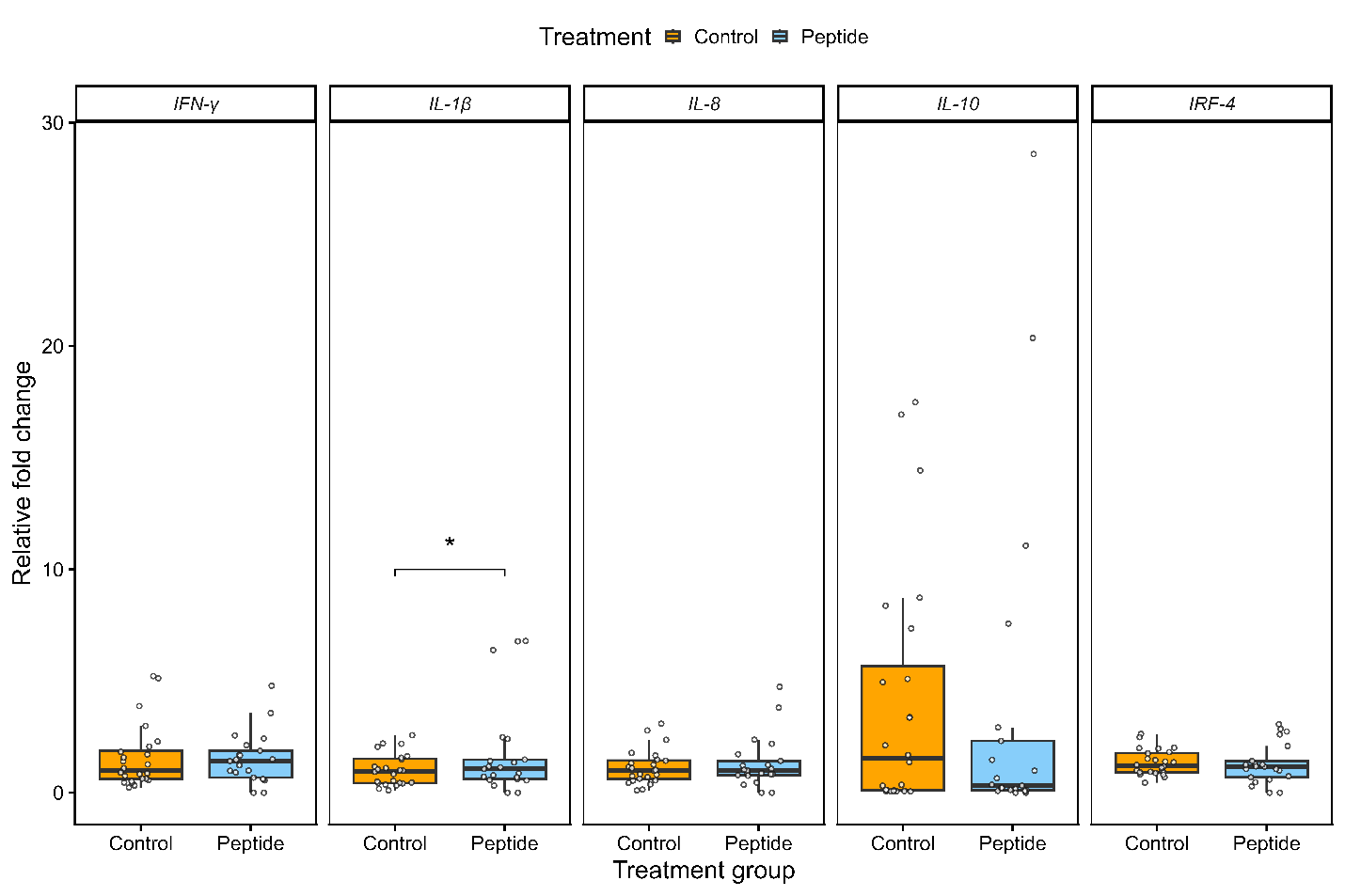


**Figure S17.** **Relative cytokine transcripts in the spleens of pen trial birds treated with 10 μg TeBi1.** Additional details in the Supplementary Figure S5 legend.


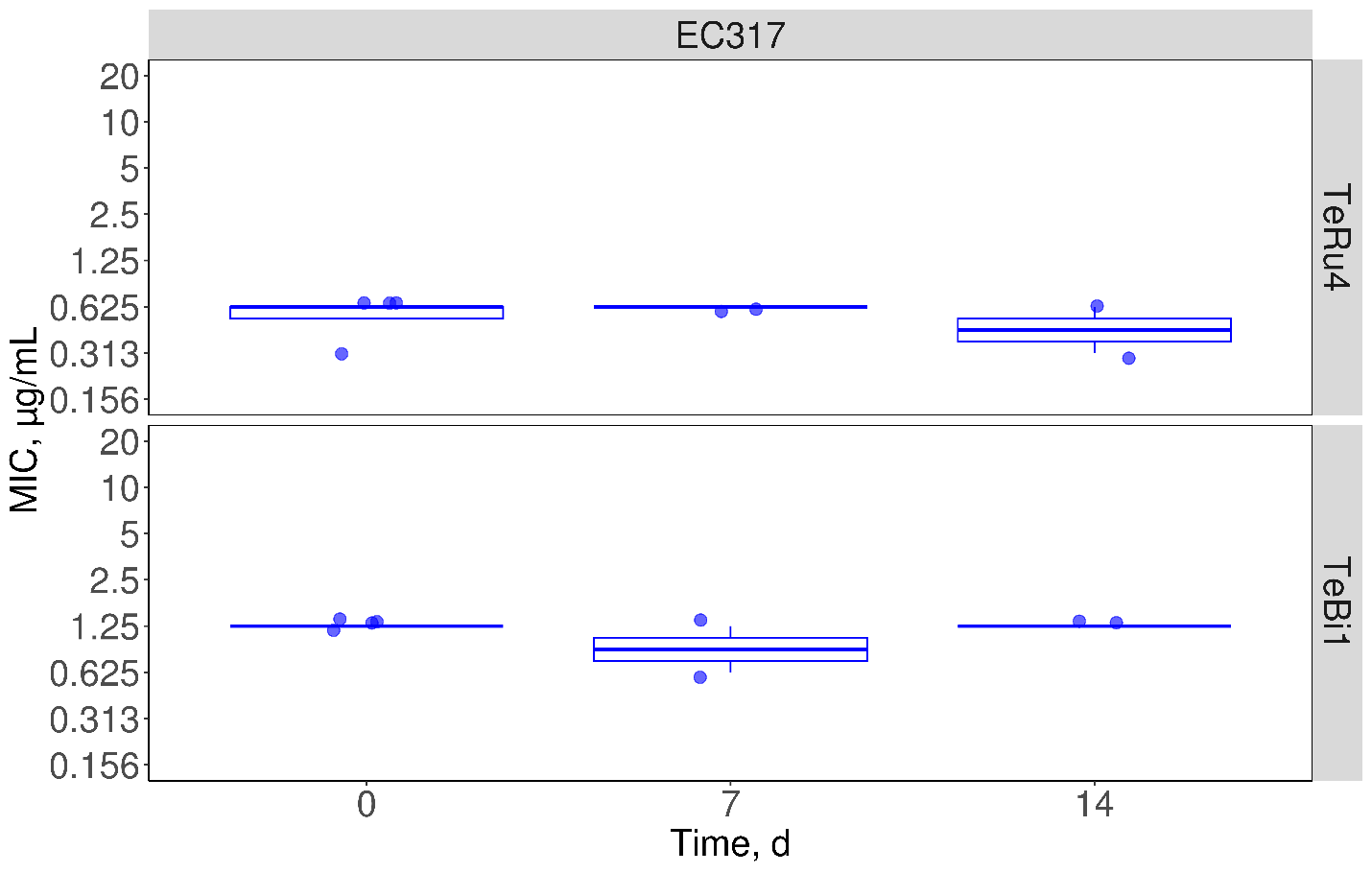


**Figure S18. Stability of TeRu4 and TeBi1 AMPs in sterile water over 14 d.** AMP solutions (2 mg/mL) were prepared in sterile water and stored at 4°C. MICs against E. coli EC317 were determined via antimicrobial susceptibility testing (AST) on d 0, 7, and 14.
