# Supplementary material for: Efficacy and safety evaluation of artificial intelligence-identified antimicrobial peptides targeting avian pathogenic Escherichia coli in broiler chickens
Source: J Anim Sci Biotechnol. 2026 May 15;17:93. doi: 10.1186/s40104-026-01417-8 (PMC13177867; doi:10.1186/s40104-026-01417-8)
Supplement: Supplementary file 2 — Additional file 2: Representative quality control data for AMPs. [file 40104_2026_1417_MOESM2_ESM.pdf]

CERTIFICATE OF ANALYSIS

**Product Name**	TeRu4
**Order ID**	U8692IA180_1
**Lot No.**	U8692IA180-1/PE4949
**Sequence**	SWLSKSVKKLVNKKNYTRLEKLAKKKLFNE
**Modification**	N/A
**Length**	30AA
**Storage**	-20°C
**Recommended Solvent***	Ultrapure water
**Comments**	TFA salt

**Test Items**	**Specifications**	**Results**
**Molecular Weight**	Theoretical MW: 3622.33	Consistent
**HPLC purity**	≥85.0%	95.7%
**Appearance**	White lyophilized powder	Conforms
**Gross Weight**	4 g	8*500.0mg

*Note: Above recommended solvents for reference only. If there is any request for detailed dissolution conditions,we suggest you choose our 'Peptide Solubility Test Service'.

Caution:
For laboratory or further manufacturing use only. Not intended for household use. If you have any questions about the Certificate of Analysis, please contact our customer service representative at 1-877-436-7274 (Toll-Free), or 1-732-885-9188.

Certified by: Ni hui Wei Date:02/11/2023

Thank you for your patronage to our Peptide services! To maintain this working relationship, we shall be grateful if you can add our webpage URL into your lab website. As a token of appreciation, you will be rewarded by 1,000 EZcoupon™ points. For more information, please contact us by e-mail at web@genscript.com

Sample Name :TeRu4
Sample ID :U8692IA180-1
Time Processed :17:43:33
Month-Day-Year Processed :02/09/2023

Pump A : 0.065% trifluoroacetic in 100% water (v/v)
Pump B : 0.05% trifluoroacetic in 100% acetonitrile (v/v)
Total Flow:1 ml/min
Wavelength:220 nm
<<LC Time Program>>

Time	Module	Command	Value
0.01	Pumps	B.Conc	5
25.00	Pumps	B.Conc	65
25.01	Pumps	B.Conc	95
27.00	Pumps	B.Conc	95
27.01	Pumps	B.Conc	5
35.00	Pumps	B.Conc	5
35.01	Controller	Stop	

<<Column Performance>>
<Detector A>
Column :Inertsil ODS-SP 4.6 x 250 mm
Equipment: ZJ19010325

<Chromatogram>

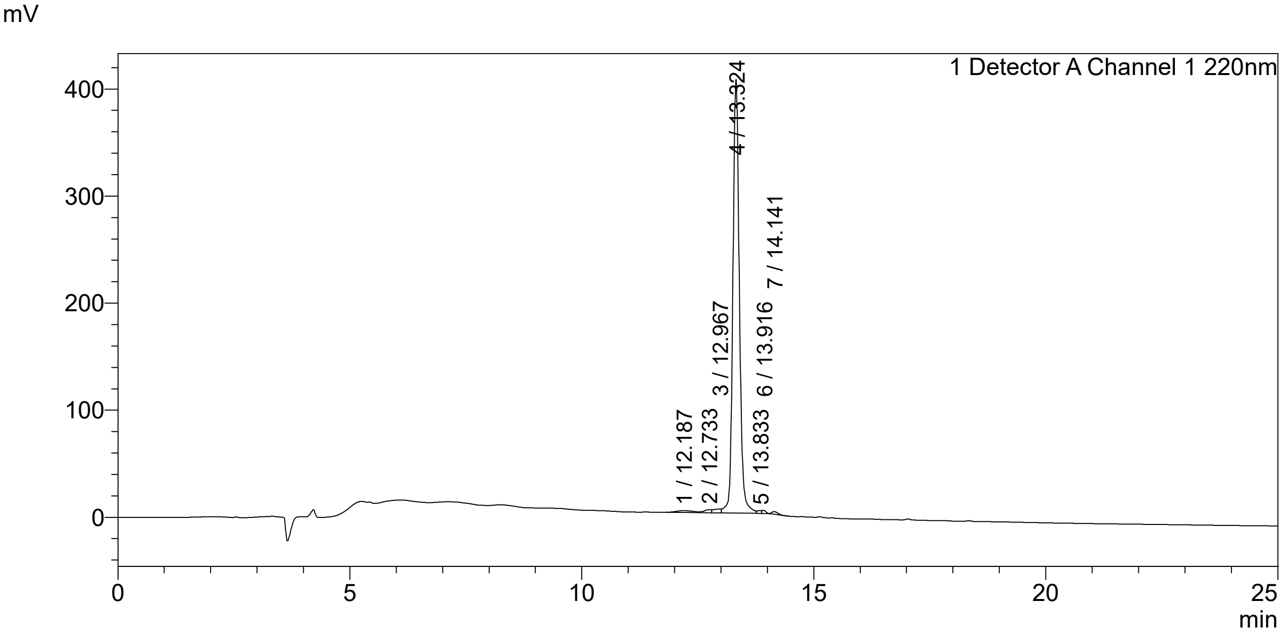

<Peak Table>

Detector A Channel 1 220nm

Peak#	Ret. Time	Area	Height	Area%
1	12.187	43524	1687	1.054
2	12.733	34737	2904	0.841
3	12.967	42815	3762	1.037
4	13.324	3953630	405130	95.719
5	13.833	17890	2717	0.433
6	13.916	16625	2898	0.402
7	14.141	21248	2381	0.514
Total		4130469	421480	100.000

Mass Spectrum

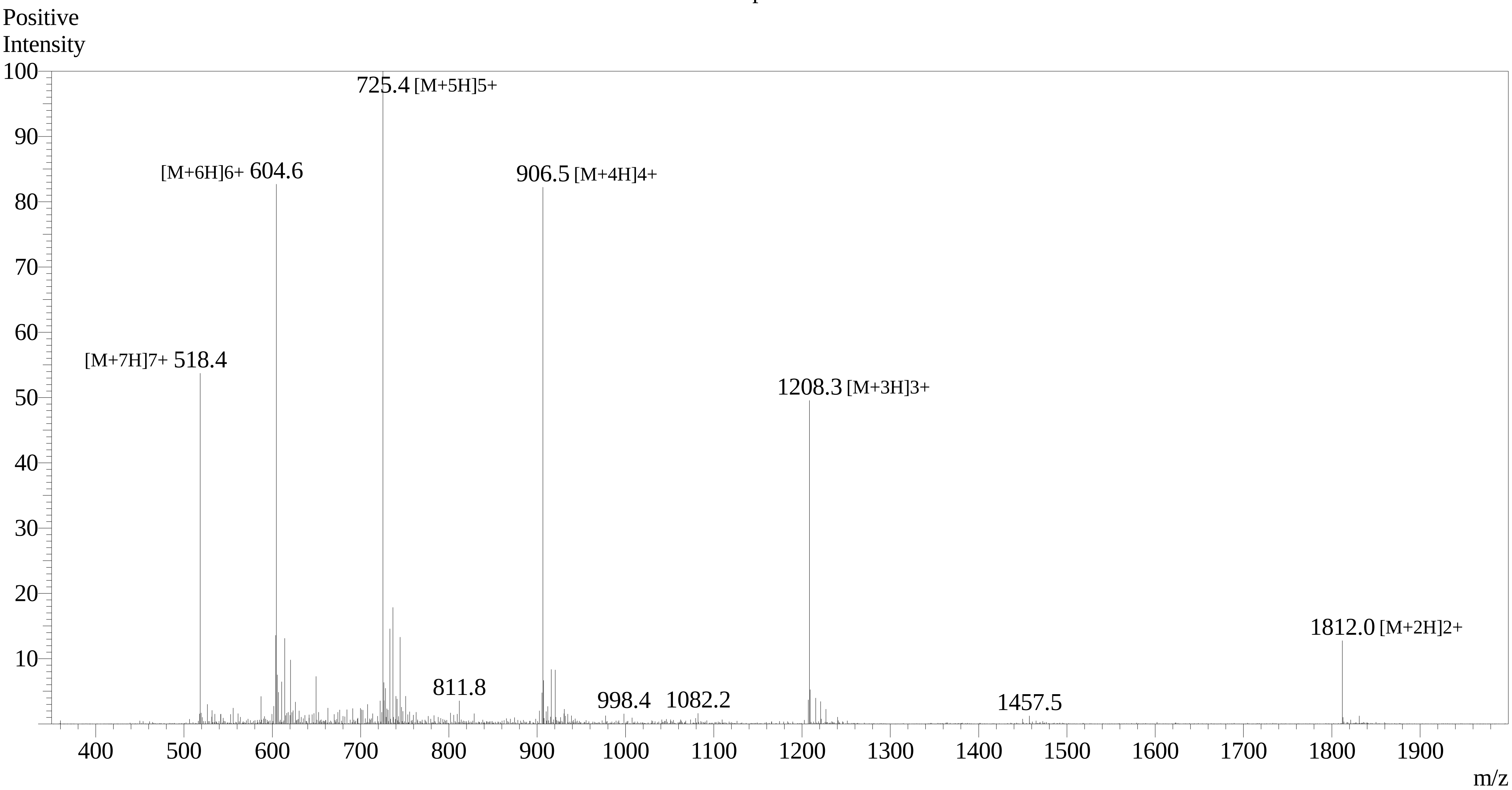

Sample Information	
Month-Day Processed :	02/08/23
Time Processed :	11:42:34
Injection Volume :	0.2
Sample Name :	TeRu4
Sample ID :	U8692IA180-1
Theoretical MW :	3622.33
Observed MW :	3622.0

Interface	:ESI
Nebulizing Gas Flow	:1.5L/min
CDL Temp	:250
Block Temp	:200

Equipment	: ZJ21010035
Interface Bias	: +4.5 kV
Drying Gas Flow	:5 L/min
T.Flow	:0.2 ml/min
B.conc	:50%H2O/50%MeOH

CERTIFICATE OF ANALYSIS

**Product Name**	TeBi1
**Order ID**	U164CARWG0_1
**Lot No.**	U164CARWG0-1/PE5001
**Sequence**	KIKIPWGKVKDFLVGGMKAVGKK
**Modification**	N/A
**Length**	23AA
**Storage***	-20°C
**Recommended Solvent***	Ultrapure water
**Comments**	TFA salt

**Test Items**	**Specifications**	**Results**
**Molecular Weight**	Theoretical MW: 2528.20	Consistent
**HPLC purity**	≥85.0%	88.0%
**Appearance**	White lyophilized powder	Conforms
**Gross Weight**	250 mg	2*125.0mg

*Note: Above recommended solvents for reference only. If there is any request for detailed dissolution conditions,we suggest you choose our 'Peptide Solubility Test Service'.

For long-term storage, it is suggested to store at -20°C and avoid freeze-thaw cycles. Since the expiration date of different peptides is different, if you are interested in more information about the expiration date, please feel free to consult the stability test service.

Caution:
For laboratory or further manufacturing use only. Not intended for household use. If you have any questions about the Certificate of Analysis, please contact our customer service representative at 1-877-436-7274 (Toll-Free), or 1-732-885-9188.

Certified by: Tong zhang Date:08/25/2024

Thank you for your patronage to our Peptide services! To maintain this working relationship, we shall be grateful if you can add our webpage URL into your lab website. As a token of appreciation, you will be rewarded by 1,000 EZcoupon™ points. For more information, please contact us by e-mail at web@genscript.com

Sample Name :TeBi1
Sample ID :U164CARWG0-1
Time Processed :14:52:12
Month-Day-Year Processed :08/24/2024

Pump A : 0.065% trifluoroacetic in 100% water (v/v)
Pump B : 0.05% trifluoroacetic in 100% acetonitrile (v/v)
Total Flow:1 ml/min
Wavelength:220 nm
<<LC Time Program>>

Time	Module	Command	Value
0.01	Pumps	B.Conc	5
25.00	Pumps	B.Conc	65
25.01	Pumps	B.Conc	95
27.00	Pumps	B.Conc	95
27.01	Pumps	B.Conc	5
35.00	Pumps	B.Conc	5
35.01	Controller	Stop	

<<Column Performance>>
<Detector A>
Column :Inertsil ODS-SP 4.6 x 250 mm
Equipment: ZJ21010376

<Chromatogram>

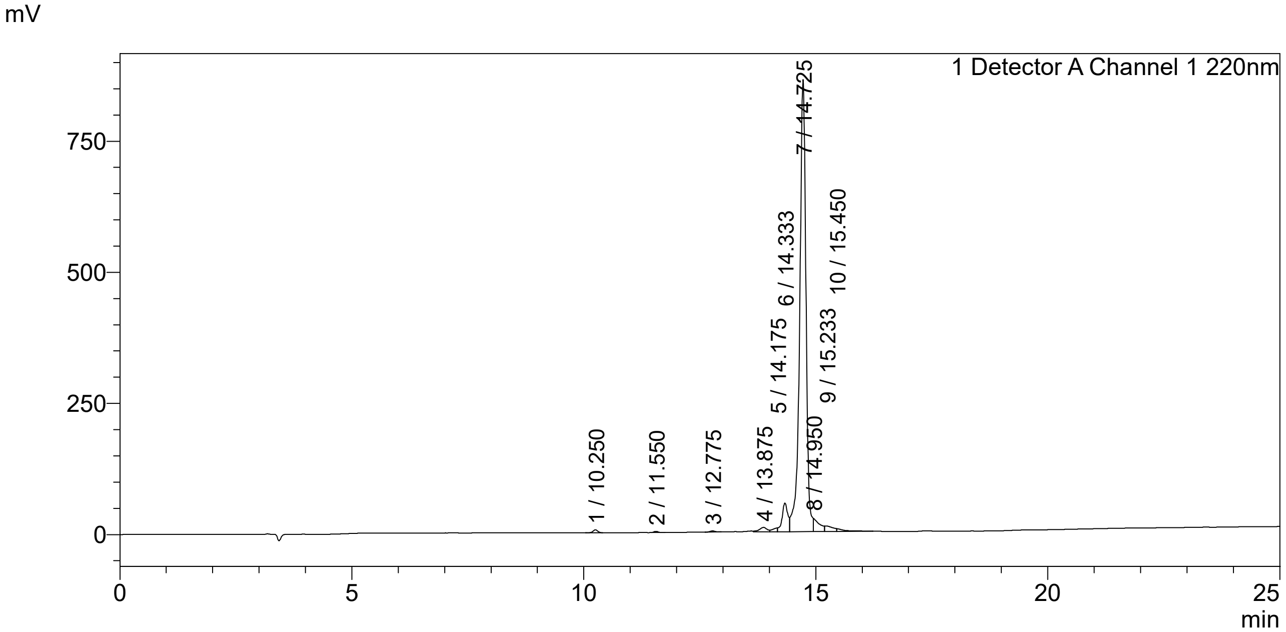

<Peak Table>

Detector A Channel 1 220nm

Peak#	Ret. Time	Area	Height	Area%
1	10.250	37128	5623	0.402
2	11.550	14639	2363	0.158
3	12.775	17060	2218	0.185
4	13.875	97121	8332	1.051
5	14.175	51416	7407	0.557
6	14.333	465219	54936	5.037
7	14.725	8125928	862290	87.973
8	14.950	230543	25391	2.496
9	15.233	128510	10369	1.391
10	15.450	69292	5367	0.750
Total		9236857	984296	100.000

Mass Spectrum

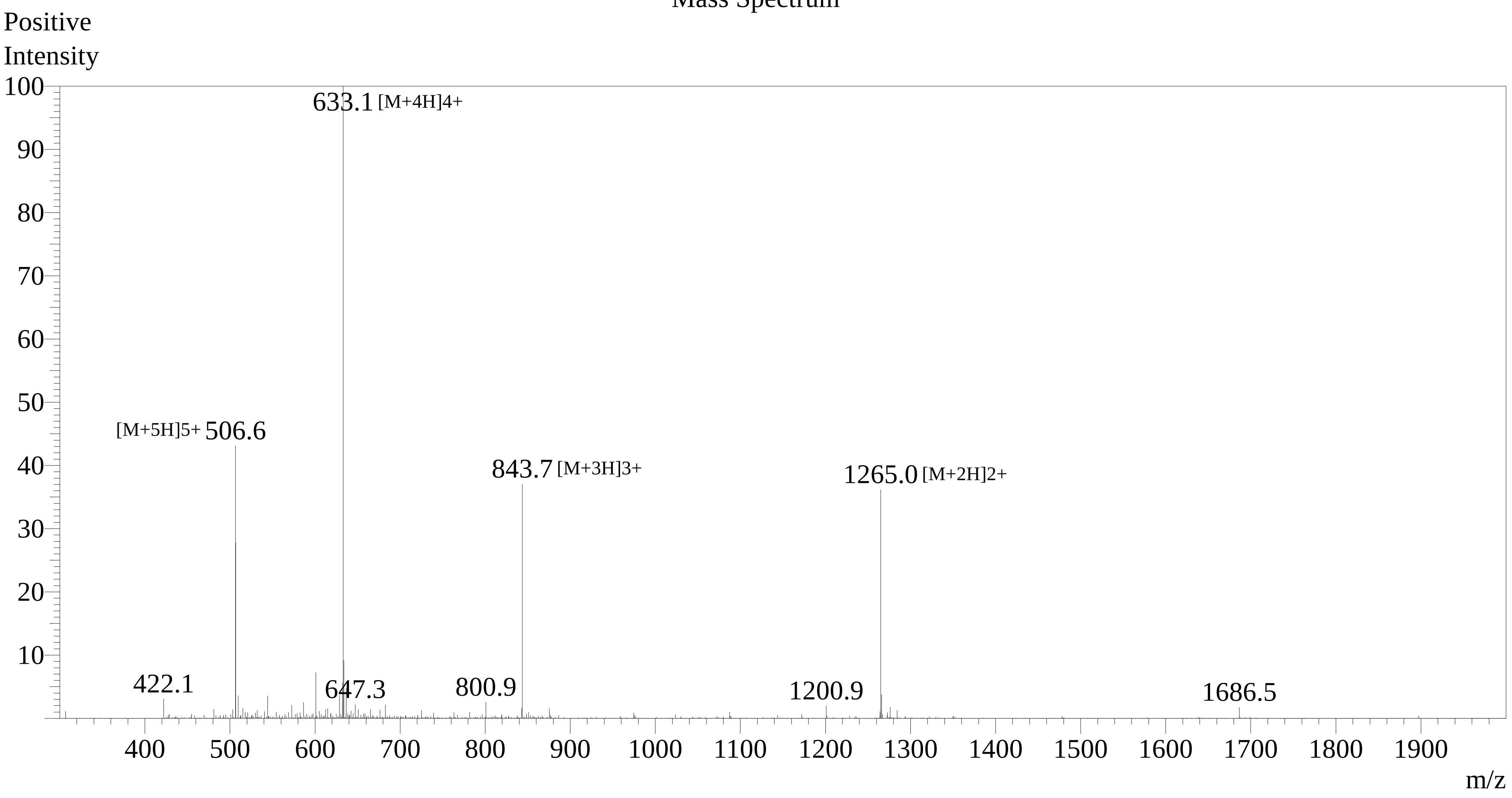

Sample Information	
Month-Day Processed :	08/23/24
Time Processed :	14:49:01
Injection Volume :	0.3
Sample Name :	TeBi1
Sample ID :	U164CARWG0-1
Theoretical MW :	2528.20
Observed MW :	2528.4

Interface	:ESI
Nebulizing Gas Flow	:1.5L/min
CDL Temp	:250
Block Temp	:200

Equipment	:ZJ22010150
Interface Bias	: +4.5 kV
Drying Gas Flow	:5 L/min
T.Flow	:0.2 ml/min
B.conc	:50%H2O/50%MeOH

CERTIFICATE OF ANALYSIS

**Product Name**	PeNi4
**Order ID**	U874XHUHG0_25
**Lot No.**	U874XHUHG0-25/PE7292
**Sequence**	GLLGKVLGVGKKVLC
**Modification**	N/A
**Length**	15AA
**Storage***	-20°C
**Recommended Solvent***	Ultrapure water
**Comments**	TFA salt

**Test Items**	**Specifications**	**Results**
**Molecular Weight**	Theoretical MW: 1483.91	Consistent
**HPLC purity**	≥85.0%	86.2%
**Appearance**	White lyophilized powder	Conforms
**Gross Weight**	500 mg	500.5mg

*Note: Above recommended solvents for reference only. If there is any request for detailed dissolution conditions,we suggest you choose our 'Peptide Solubility Test Service'.

For long-term storage, it is suggested to store at -20°C and avoid freeze-thaw cycles. Since the expiration date of different peptides is different, if you are interested in more information about the expiration date, please feel free to consult the stability test service.

Caution:
For laboratory or further manufacturing use only. Not intended for household use. If you have any questions about the Certificate of Analysis, please contact our customer service representative at 1-877-436-7274 (Toll-Free), or 1-732-885-9188.

Certified by: Yi Gao Date:02/29/2024

Thank you for your patronage to our Peptide services! To maintain this working relationship, we shall be grateful if you can add our webpage URL into your lab website. As a token of appreciation, you will be rewarded by 1,000 EZcoupon™ points. For more information, please contact us by e-mail at web@genscript.com

Sample Name : PeNi4
Sample ID : U874XHUHG0-25
Time Processed :13:00:32
Month-Day-Year Processed :02/27/2024

Pump A : 0.065% trifluoroacetic in 100% water (v/v)
Pump B : 0.05% trifluoroacetic in 100% acetonitrile (v/v)
Total Flow:1 ml/min
Wavelength:220 nm
<<LC Time Program>>

Time	Module	Command	Value
0.01	Pumps	B.Conc	5
25.00	Pumps	B.Conc	65
25.01	Pumps	B.Conc	95
27.00	Pumps	B.Conc	95
27.01	Pumps	B.Conc	5
35.00	Pumps	B.Conc	5
35.01	Controller	Stop	

<<Column Performance>>
<Detector A>
Column :Inertsil ODS-SP 4.6 x 250 mm
Equipment: GR11010440

<Chromatogram>

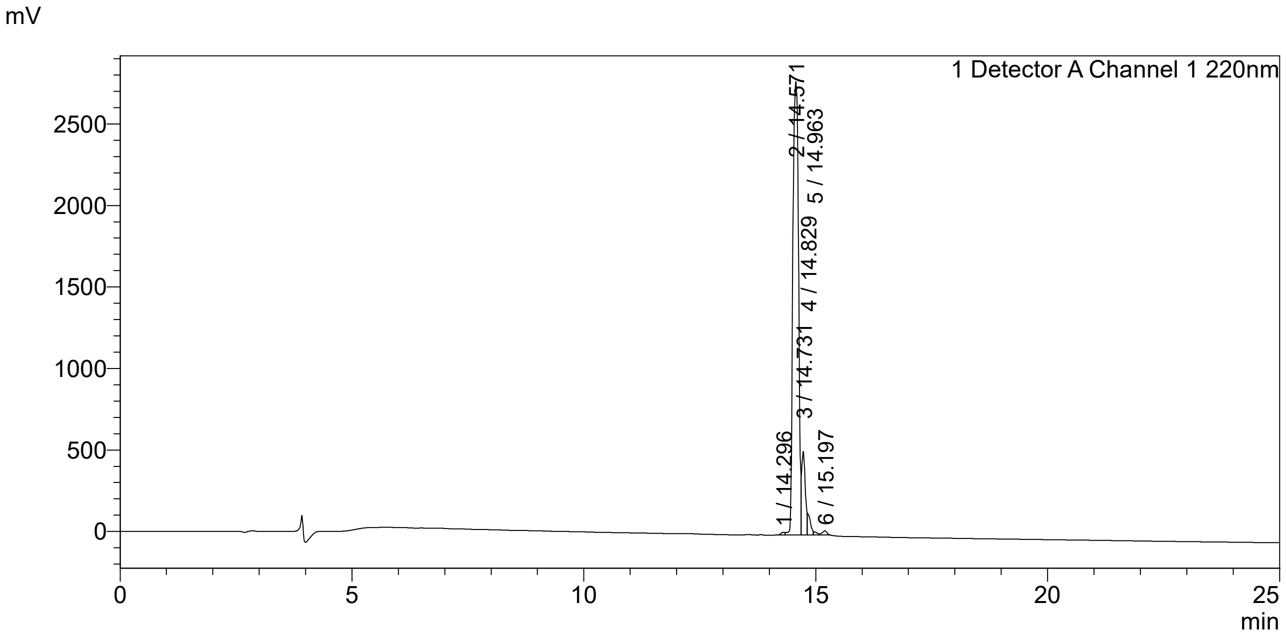

<Peak Table>

Detector A Channel 1 220nm

Peak#	Ret. Time	Area	Height	Area%
1	14.296	97132	16337	0.355
2	14.571	23566722	2780999	86.221
3	14.731	2795622	512227	10.228
4	14.829	604136	130397	2.210
5	14.963	98240	18768	0.359
6	15.197	171172	24582	0.626
Total		27333024	3483309	100.000

Mass Spectrum

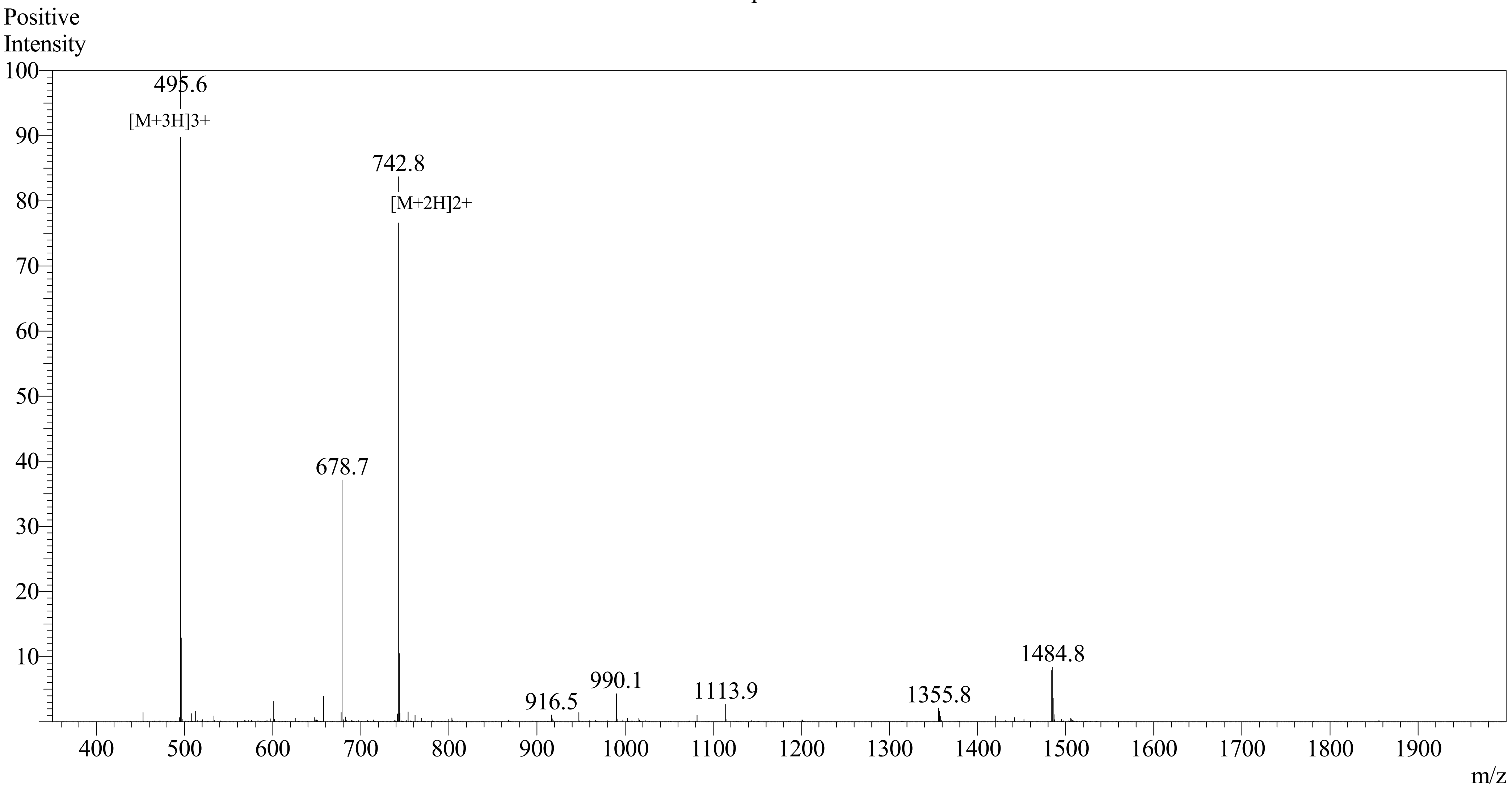

Sample Information	
Month-Day Processed :	02/26/24
Time Processed :	8:47:12 AM
Injection Volume :	0.3
Sample Name :	PeNi4
Sample ID :	U874XHUHG0-25
Theoretical MW :	1483.91
Observed MW :	1483.8

Interface	:ESI
Nebulizing Gas Flow	:1.5L/min
CDL Temp	:250
Block Temp	:200

Equipment	:ZJ22010150
Interface Bias	: +4.5 kV
Drying Gas Flow	:5 L/min
T.Flow	:0.2 ml/min
B.conc	:50%H2O/50%MeOH
